# Predictions of Through-Focus Performance of Presbyopia-Correcting Intraocular Lenses in Presbyopic Subjects Using a Visual Simulator

**DOI:** 10.1016/j.xops.2026.101140

**Published:** 2026-02-28

**Authors:** Petros Papadogiannis, Xoana Barcala, Irene Siso-Fuertes, Amal Zaytouny, Lucie Sawides, Carlos Dorronsoro, Susana Marcos

**Affiliations:** 1 2EyesVision S.L., Madrid, Spain; 2 Institute of Optics, Spanish National Research Council, IO-CSIC, Madrid, Spain; 3 Center for Visual Science, The Institute of Optics, Flaum Eye Institute, University of Rochester, New York

**Keywords:** Multifocal intraocular lenses (MIOLs), Extended depth of focus intraocular lenses (EDOFs), Visual simulation, Preoperative prediction, Subject-specific visual outcomes

## Abstract

**Purpose:**

To validate whether visual simulations of multifocal intraocular lenses and extended depth of focus (EDOF) intraocular lenses (IOLs) in phakic presbyopes replicate postoperative vision, and to compare performance across these lenses in the same subject.

**Design:**

A cross-sectional study; evaluation of technology.

**Subjects:**

Twenty-three presbyopes with clear crystalline lenses were evaluated binocularly using SimVis Gekko visual simulator (2EyesVision).

**Methods:**

Six commercial multifocal and EDOF IOLs were simulated using the principle of temporal multiplexing: AcrySoft IQ PanOptix, FineVision POD F, AT Lisa tri, Tecnis Symfony, AcrySoft IQ Vivity, and AT Lara. Defocus visual acuity (DFVA) curves were measured for each simulated IOL under photopic conditions. Simulated IOLs were programmed from published through-focus modulation transfer function data at 15 cycles/degree. Defocus visual acuity curves through simulated IOLs were compared to average postoperative DFVA data from peer-reviewed publications (108–322 pseudophakic patients per IOL).

**Main Outcome Measures:**

Cross-correlation, root mean square error (RMSE), depth of focus (DOF), and relative visual benefit/degradation at far, intermediate, and near distances.

**Results:**

Simulated IOLs accurately reproduced on-bench optical profiles in terms of through-focus Visual Strehl Ratio (cross-correlation >0.98, RMSE <0.04). Defocus visual acuity curves through simulated IOLs closely matched postoperative curves in subjects implanted with the corresponding IOL (cross-correlation ≥0.94, RMSE ≤0.07), with far vision differences ≤0.09 logarithm of the minimum angle of resolution (logMAR). Discrepancies were largest at high near vergences (up to 0.17 logMAR). Trifocal IOLs (PanOptix, FineVision, AT Lisa) expanded DOF by ∼1.5 D compared with monofocal IOLs, while EDOF IOLs (Symfony, Vivity, AT Lara) expanded DOF by ∼0.7 D. Visual simulations replicated relative gains at intermediate/near and degradations at far, consistent with clinical outcomes in implanted patients. Intersubject variability observed in simulations mirrored that reported postoperatively.

**Conclusions:**

The SimVis Gekko visual simulator reproduces, in a simulated preimplantation context, the reported postoperative visual performance, both with multifocal and EDOF IOLs, capturing both group-level and subject-specific variability. The results suggest that this tool can provide patients with a representative preview of relative visual performance of different IOLs prior to surgery.

**Financial Disclosure(s):**

Proprietary or commercial disclosure may be found in the Footnotes and Disclosures at the end of this article.

Multifocal intraocular lenses (MIOLs) and extended depth of focus (EDOF) intraocular lenses (IOLs) aim at giving patients extended near vision and vision functionality while preserving far vision. The operation of these lenses relies on the principle of simultaneous vision, where projected focused and defocused images at various distances are superimposed on the retina.^1^ The functionality across distances is therefore not achieved by dynamic focusing (as in the young crystalline lens, before presbyopia onset) but rather by a static optical expansion of the range of focus, either providing distinct foci at far, intermediate and distance, or a smoother transition between zones. Multifocal intraocular lenses providing 2, or recently, most commonly, 3 foci, are generally based on diffractive optics principles. Examples of diffractive trifocal IOLs currently available in the market include the AcrySoft IQ PanOptix by Alcon, the FineVision POD F by BVI, the Tecnis Synergy by Johnson and Johnson, and the AT Lisa Tri by Zeiss.^2^ Designs that, rather than providing foci at discrete planes, aim at a constant quality over a certain range of focus, generally shorter than provided by MIOLs, are generally grouped in the EDOF category.^3^^,^^4^ Some manufacturers identify EDOF IOLs that achieve EDOF through either diffractive principles, for example, tuning the design parameters of a diffractive bifocal lens such that different wavelengths focus on different planes between the 2 main foci (Symphony by Johnson and Johnson), or by creating a radially localized phase shift (Vivity by Alcon). More conventionally, EDOF is created by refractive principles, using high order conic surfaces to maximize optical quality in an axial range (Isopure by BVI)^5^ or tuning the spherical aberration (Eyhance by Johnson and Johnson).^6^

The optical performance of presbyopia correction IOLs is generally evaluated theoretically by computer simulations or experimentally on bench using artificial eye models.^7^, ^8^, ^9^ While those models are useful in lens design and as quality control of the manufacturing, they do not completely relate to patient's individual visual performance. In order to evaluate the functional quality of IOLs in patients, visual tests that capture the interaction of the IOL design with the patient's optics and their neural function are required.

Defocus visual acuity (DFVA) curves are routinely used to assess visual performance in patients with implanted IOLs. In this method, different vergences are introduced, for instance, with trial lenses or a Badal optometer,^10^, ^11^, ^12^ and high-contrast visual acuity (VA) is measured at each distance. Measuring DFVA curves is time consuming and is limited to a specific function, but to date, this approach represents the most comprehensive assessment of the performance of presbyopic corrections available in the clinical practice, and it is considered a gold standard in the scientific literature, patents, and regulatory reports.^13^, ^14^, ^15^ Defocus VA curves describe visual performance in a certain range of focus, and they are also used to estimate depth of focus (DOF).^16^, ^17^, ^18^ Many reports compare postoperative DFVA curves across different designs.^19^ However, in pseudophakic patients, such comparisons can only be made between separate groups of patients implanted with different lenses, because—unlike with contact lens correction—each patient can only be evaluated with 1 implanted design at a time.

In contrast, visual simulators of presbyopic corrections allow the same patient, preoperatively, the experience of prospective postoperative vision with different IOL designs.^20^, ^21^, ^22^ Because these devices are programmable and noninvasive, patients can assess vision through simulated optics without wearing contact lenses and before IOL implantation. Visual simulators thus capture the interaction of the IOL design with the patient's ocular and neural component, and—unlike postoperative evaluation—enable direct comparison of the visual performance across different IOLs.

In prior work, we reported DFVA measured through lenses programmed in on-bench adaptive optics (AO) visual simulators and in simultaneous vision simulators.^7^ In AO visual simulators, a phase map representation of the IOL is mapped on a spatial light modulator (for diffractive and segmented profiles) or on a deformable mirror (smooth varying profiles).^7^^,^^23^^,^^24^ An alternative to spatial representation of the lens is temporal multiplexing (SimVis), in which lenses are simulated by rapidly cycling lens power to reproduce the through-focus performance of the IOL.^25^ In the former approaches, VA stimuli are typically displayed on a monitor or mini display within the device, limiting the field of view to about 2°. The latter, a see-through device, provides a wider field of view (20°), enabling patients to view natural scenes and standard clinical eye charts, as well as to undergo binocular measurements.^18^^,^^22^

Several studies have validated the fidelity of the visual simulations, through cross-comparisons of AO-based, temporal multiplexing-based, and physical IOLs immersed in a cuvette and projected onto the eye.^12^^,^^22^ Comparisons of DFVA through simulated contact lenses and actual contact lenses on eye likewise show strong correspondence.^11^^,^^26^^,^^27^ In all such studies, the crystalline lens was present across conditions, confirming the accuracy of the programmed simulation. However, applying these methods preoperatively to predict postoperative vision is only valid as long as the contribution of the crystalline lens (present preoperatively but absent postoperatively) is minimal.

Villegas et al^28^ used an AO visual simulator to show that the spherical aberration of the eye, particularly crystalline lens aberrations, minimally affect simulated performance. Similarly, Vinas et al^29^ studied a small cohort of patients measured both preoperatively with SimVis (simulating the FineVision POD F diffractive trifocal IOL, BVI) and postoperatively with the implanted IOL, finding strong correspondence between preoperative and postoperative DFVA. In eyes with clear crystalline lenses, preoperative and postoperative DFVA curves matched in both shape and absolute VA values. In eyes with cataracts, the curves matched in shape, but the preoperative curves were shifted downward (worse through-focus VA) across distances, reflecting the scattering effect of the cataract. With spatial mapping simulators, opacities may disrupt the simulation by blocking areas of the lens devoted to particular focal distances. By contrast, temporal multiplexing projects the simulated lens effects directly onto the retina, bypassing opacities; while overall contrast is reduced, the relative through-focus performance is preserved. In line with these findings, Barcala et al^30^ evaluated vision with 4 different IOL simulations in patients both before cataract surgery (with an opacified crystalline lens) and after implantation of a monofocal IOL. The perceptual ranking of designs was consistent preoperatively and postoperatively, although scores were higher postoperatively, as expected.

Providing patients with a preoperative experience of postoperative correction addresses an important clinical need: managing expectations and guiding IOL selection. Selecting the optimal IOL has become increasingly challenging with the growing number of presbyopia-correcting IOLs available. The success of the simulations depends on their ability to reproduce the postoperative performance of the real IOLs. In a recent pilot study, Sawides et al^31^ used a commercially available temporal multiplexing visual simulator (SimVis Gekko) to simulate 3 IOLs (Tecnis Symfony, AcrySoft IQ PanOptix, FineVision POD F). In that work, the lenses were programmed using published on-bench performance data, and DFVA curves obtained in subjects looking through the simulated IOLs were compared with literature-reported postoperative DFVA curves from patients implanted with those same lenses. That study demonstrated the feasibility of generating realistic preoperative visual simulations from publicly available data, but it was limited to 3 IOLs and a small number of subjects. In this study, we extend this approach by simulating 6 commercially available IOL designs from 4 manufacturers in 23 presbyopic clear-lens subjects. The simulations were based solely on publicly available through-focus modulation transfer functions (MTFs) obtained from bench-evaluating the physical IOLs. The simulated outcomes were benchmarked against reported through-focus VA from 33 published studies of patients implanted with the same lenses, encompassing sample sizes ranging from 108 to 322 patients per IOL. Beyond replicating on-bench optical profiles, our study provides a quantitative evaluation of the simulations including cross-correlation, root mean square error (RMSE), DOF and relative visual benefit, and degradation at far, intermediate, and near distances. This approach allows us to assess both group-level and subject-specific correspondence between simulated and actual postoperative vision, providing broader and more robust validation than previously reported.

## Methods

Binocular DFVA curves were measured in clear lens presbyopic subjects through a binocular visual simulator (manufactured by 2EyesVision) operating under SimVis technology. The following 6 commercial MIOLs were simulated: AcrySoft IQ PanOptix (Alcon), FineVision PODF (BVI), AT Lisa tri (ZEISS), Tecnis Symfony (Johnson & Johnson), AcrySoft IQ Vivity (Alcon), and AT Lara (ZEISS). For each IOL, binocular-simulated DFVA curves were compared with averaged DFVA reported in the literature from patients implanted with the same commercial designs. Table 1 summarizes the characteristics of the IOLs in study (model, manufacturer, type, optical technology, power for far, intermediate, and near addition).^32^, ^33^, ^34^

**Table 1:** Characteristics of the IOLs under Study∗

IOL Model	Manufacturer	Type	Optical Technology	Power (Far)	Additions (Intermediate:I, Near:N)
PanOptix	Alcon Laboratories Inc	Trifocal	Diffractive	+20.5 D^32^	I: +2.17 D, N: +3.25 D^2^
FineVision	BVI Medical Inc/PhysIOL	Trifocal	Diffractive	+19.5 D^32^	I: +1.75 D, N: +3.5 D^32^
ATLisa Tri	Carl Zeiss Meditec	Trifocal	Diffractive	+22.0 D^32^	I: +1.66 D N: +3.33 D^2^
Symfony	Johnson & Johnson Surgical Vision	EDOF	Diffractive	+20.0 D^32^	I: +1.75 D^33^
Vivity	Alcon Laboratories Inc	EDOF	Refractive	+20.0 D^34^	I: +1.53 D^2^
ATLara	Carl Zeiss Meditec	EDOF	Diffractive	+20.0 D^21^	I: +1.9 D^17^

IOL = intraocular lens.

∗ Bench optical data sources listed in Table 3.

### Subjects

Twenty-three clear lens presbyopic subjects with normal ocular health were recruited at the Visual Optics and Biophotonics Lab (CSIC) to participate in the study. Exclusion criteria included history of ocular disease, eye surgery, and lens opacities. Baseline measurements included slitlamp imaging to rule out the presence of opacification, refraction, best-corrected distance binocular VA, and required near add. Noncyclopegic refraction was measured with the Nidek ARK-1 autorefractometer. Best-corrected binocular logarithm of the minimum angle of resolution (logMAR) VA at distance was measured under photopic conditions. A high-contrast ETDRS chart presented on a TV screen with a luminance of ≈85 cd/m^2^ connected to Optonet optometric software^35^ was used. The TV was located 4 m from the subject. The required near add was determined by using the age rule table as a starting point and fine-tuning the near add based on the subject's performance on reading. Measurements were performed in 2 different sessions, during which different lenses were evaluated. In session I, simulations of the Tecnis Symfony, AcrySoft IQ PanOptix, and FineVision PODF IOLs were evaluated. In session II, simulations of the AcrySoft IQ Vivity, AT Lisa tri, and AT Lara were evaluated. A total of 8 subjects participated in session I only; a total of 8 subjects participated in session II only; and 7 subjects participated in sessions I and II. Therefore, each lens was evaluated in 15 subjects, whose characteristics are presented in Table 2.^33^

**Table 2:** Characteristics of the Subjects

Subjects		
	Session I	Session II
Age (yrs)	55.5 ± 5.2	55 ± 5.4
Spherical refractive error (D)	Range: –4.00 to +4.00	Range: –6.50 to +4.00 D
Cylindrical refractive error (D)	Less than –1.25	Less than –2.50
Average best-corrected binocular visual acuity at far (logMAR)	–0.10 ± 0.01	–0.10 ± 0.01
Average near addition (D)	1.93 ± 0.35 (Range: +1.50–+2.50)	1.92 ± 0.37 (Range: +1.50–+2.50)

D = diopter; logMAR = logarithm of the minimum angle of resolution.

Protocols were approved by the CSIC Ethics Committee. The experiments conformed to the tenets of the Declaration of Helsinki. Prior to any measurement, subjects signed an informed consent after receiving an explanation of the nature and implications of the study.

### SimVis-Based Lens Performance: Calculations and Experimental Validation

The simulated lenses are programmed in SimVis for a 3-mm pupil diameter (in the IOL plane) and simulated using publicly available IOL through-focus MTF (TF-MTF) curves at 15 cycles/degree (equivalent to 50lp/mm), following methodology described in detail by Sawides et al.^36^

#### Through-Focus MTF at 15 Cycles/Degree from Literature

First, the IOL TF-MTF curves at 15 cycles/degree, for a 3-mm pupil diameter, are extracted from scientific publications or regulatory documents and normalized by a factor corresponding to the MTF diffraction-limited (for similar spatial frequency and pupil diameter). When the TF-MTF curves were available for 3 wavelengths, the average TF-MTF weighted by the luminous efficiency function^37^ (0.11, 0.99, and 0.15 for red, green, and blue, respectively) is calculated and then normalized.

#### Estimation of TF-VA

A zero-phase digital filter is then applied to smooth the curve where a low-pass finite impulse response digital filter (optimized using different synthetic lenses of different design types)^36^ is used, with the following design parameters and options: pass band frequency 0.05 D^–1^, stop band frequency from 0.5 D^–1^, pass band ripple 0.05 dB, stop band attenuation 10 dB, and design method Kaiser window. The filtered signal represents the estimated through-focus visual Strehl ratio (TFVS) in the IOL plane and converted to the entrance pupil plane,^38^ where SimVis^39^ technology simulates the IOL. Table 3 lists the scientific publications or regulatory documents from which the data are extracted.

**Table 3:** Publicly Available Data of the 6 IOLs Used in the Study

IOL Model	SimVis Simulation Name	Reference Paper	Fig.	Wavelengths (nm)	TF-MTF Measurement Equipment	Corneal Model
PanOptix	PanOp	Loicq et al, 2019^32^	11a	480/546/650	PMTF, lambda-X	SA-free
FineVision	FineV	Loicq et al, 2019^32^	9a	480/546/650	PMTF, lambda-X	SA-free
ATLisa	ATLis	Loicq et al, 2019^32^	10a	480/546/650	PMTF, lambda-X	SA-free
Symfony	Symfo	Loicq et al, 2019^32^	7a	480/546/650	PMTF, lambda-X	SA-free
Vivity	Vivit	FDA document^34^	4	White light (taken as 555)	N/A	N/A
ATLara	ATLar	Chae et al, 2020^39^	1c	545	PMTF, lambda-X	SA-free

IOL = intraocular lens; PMTF = power and modulation transfer function; SA = spherical aberration; TF-MTF = through-focus modulation transfer function.

#### Estimation of Temporal Coefficients

Then the SimVis TFVS is obtained and validated using the methodology previously reported in detail.^12^^,^^25^^,^^31^^,^^40^ In brief, from the estimated TFVS, a temporal profile is obtained consisting in a set of temporal coefficients calculated following the equations proposed by Akondi et al, and optimized in an iterative procedure, indicating the relative time in a SimVis cycle (20 ms) that the tunable lens must stay at each additions (in 0.05 D steps) such that the SimVis TFVS mimics the estimated TFVS under temporal multiplexing at 50 Hz. Mathematically, these temporal coefficients represent the weighting factors of a series of monofocal point spread functions for different optical power (additions) tuned to match the through-focus performance of the IOL under study (estimated TFVS). The number of temporal coefficients varies with IOL design. Table 4 shows the number of temporal coefficients required in the SimVis simulations of each of the IOLs in the study.

**Table 4:** Number of Temporal Coefficients Needed for the Simulation of Each IOL on Study

Model	# Temporal Coefficients
PanOptix	7
FineVision	4
ATLisa	6
Symfony	8
Vivity	6
ATLara	4

IOL = intraocular lens.

#### Experimental Characterization of Simulated IOL Using High-Speed Focimetry

Finally, the SimVis simulations are validated using a high speed focimeter, as described in previous publications,^40^^,^^41^ which consisted of measuring the response of the optotunable lens to the temporal profile of the SimVis simulation to obtain the Experimental SimVis TFVS, mimicking the SimVis TFVS (peaks shift less than one-fifth of a diopter, RMSE <0.05) in the defocus range of interest. Then, the simulated lens profiles are uploaded in the controlling device of the SimVis Gekko (2EyesVision), a binocular see-through headset,^42^^,^^43^ through which the visual measurements are performed, as described in previous publications.^31^

### DFVA Curves with SimVis Simulated IOLs

A study session consisted of a run of measurements of binocular DFVA curves through 3 different simulated lenses using a commercial version of the SimVis technology, the SimVis Gekko. The order of simulated lenses within each session was randomized across subjects. Measurements were conducted by an experienced device user, who mounted the device on the subject's head, aligned the oculars to the pupils of the eyes, and inserted trial lenses with the subject's sphero-cylindrical refraction (adjusted for vertex distance if needed) in the dedicated slots on the SimVis Gekko device.

**Figure 1:**
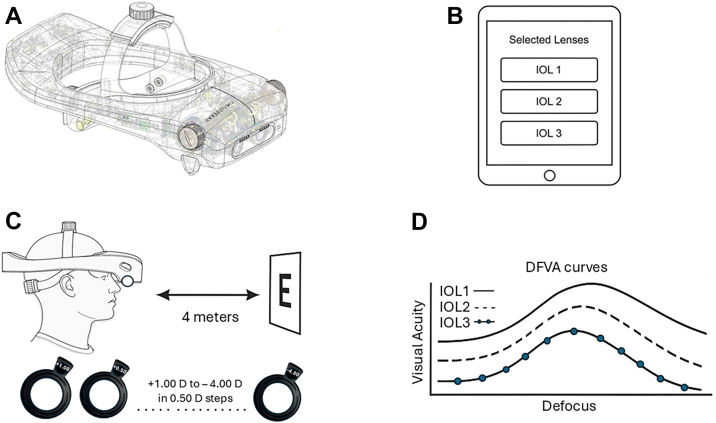
Experimental apparatus: **(A)** SimVis Gekko; **(B)** tablet with which SimVis Gekko is controlled wirelessly; **(C)** SimVis Gekko is adjusted on subject's head to perform the through-focus visual acuity evaluation utilizing trial lenses; **(D)** defocus curves (DFVA) obtained after vision evaluation. DFVA = defocus visual acuity; IOL = intraocular lens.

Subsequently, DFVA curves were obtained binocularly in a defocus range from –4.00 D to +1.00 D around the best focus in 0.50 D steps.^44^ Stimuli were presented under photopic conditions on a high-contrast ETDRS optotype chart (TV screen with a luminance of ≈85 cd/m^2^ connected to Optonet), located 4 m from the subject. The defocus level was modified with trial lenses, inserted manually in the dedicated slot for trial lenses, in a randomized sequence. The optotypes were randomly changed in each defocus step to avoid bias from learning effects. Figure 1 presents the experimental apparatus to acquire the DFVA curves.

### Literature DFVA Curves

Defocus VA curves from real patients implanted with the IOLs under study were obtained from the literature. Published results were compiled from a total of 33 publications, totaling 2 to 8 publications per IOL design. The reference DFVA curve for a given IOL was obtained as the average of pooled data. Visual acuity values were extracted point by point directly from the graphs using semiautomated MATLAB algorithms. Each graph was imported into MATLAB, and custom script calibrated the axes by manually selecting 2 reference points with known coordinate values. Data points along each curve were then manually selected with a crosshair cursor, and the script converted their positions into values using the established calibration. This approach enabled accurate digitization of the graphical data, while minimizing systematic errors associated with manual extraction.

Table 5 lists the publications used to establish the reference DFVA curves on patients. These papers were selected using the following criteria: (1) reports published after 2018; (2) minimum sample size of 10 patients; (3) report results of distance-corrected binocular DFVA curves; (4) data reported at ≥1 month postoperatively; and (5) same measurement protocols (photopic conditions, use of ETDRS chart). Table 5 also provides details on the patient cohort and measurement conditions in each of the studies. The PanOptix IOL reference curve is based on 7 publications^45^, ^46^, ^47^, ^48^, ^49^, ^50^, ^51^ with data from 322 pseudophakic patients (66.54 ± 2.98 years old). The FineVision IOL is based on 5 publications^48^^,^^50^, ^51^, ^52^, ^53^ with data from 198 patients (62.0 ± 7.13 years old). The ATLisa reference curve is based on 7 publications^19^^,^^48^^,^^54^, ^55^, ^56^, ^57^, ^58^ with data from 309 patients (61.42 ± 3.58 years old). The Symfony IOL reference curve is based on 8 publications^19^^,^^45^^,^^48^^,^^50^^,^^52^^,^^59^, ^60^, ^61^ with data from 188 patients (67.38 ± 4.18 years old). The Vivity IOL reference curve is based on 4 publications^62^, ^63^, ^64^, ^65^ with data from 202 patients (70.25 ± 8.42 years old), and the ATLara reference curve is based on 2 publications^66^^,^^67^ with data from 108 patients (65.20 ± 0.28 years old).

**Table 5:** Literature Used for Establishing the Patients’ Reference for DFVA Curves

IOL	Literature References	Figure in Paper	# Patients	Follow-Up Time (Mos)	Average Age
PanOptix	Farvardin et al,_2021^45^	1	20	3–18	62.00 ± 5.00
	Galvis et al,_2022^46^	1	65	12	67.2 ± 11.00
	Imburgia et al,_2021^47^	3	16	12	69.70 ± 8.00
	Palomino-Bautista et al,_2021^48^	2	25	3	64.80 ± 3.00
	Kohnen et al,_2020^49^	5	145	12	68.90 ± 9.00
	Rementería-Capelo et al,_2021^50^	3	36	3	69.20 ± 6.00
	Ribeiro and Ferreira,_2020^51^	3	15	3	64.00 ± 6.00
FineVision	Paik et al,_2020^52^	3	20	6	56.70 ± 7.00
	Rementería-Capelo et al,_2021^50^	3	32	3	71.13 ± 8.00
	Kim et al,_2022^53^	2	106	3	54.90 ± 5.00
	Palomino-Bautista et al,_2021^48^	2	25	3	59.50 ± 2.00
	Ribeiro and Ferreira,_2020^51^	3	15	3	68.00 ± 8.00
AT Lisa	Gil et al,_2020^19^	2	19	6	60.68 ± 7.00
	Lapid-Gortzak et al,_2020^54^	3	81	4–6	65.60 ± 9.60
	Lubiński et al,_2020^55^	2	20	12	55.00 ± 7.10
	Piovella et al,_2019^56^	1b	114	3	63.70 ± 8.70
	Palomino-Bautista et al,_2021^48^	2	25	3	60.68 ± 3.00
	Shen et al,_2022^57^	2	30	3	59.80 ± 9.00
	Acar and Nurozler Tabakci,_2021^58^	2	20	6	64.50 ± 6.00
Symfony	Rementería-Capelo et al,_2021^50^	3	23	3	65.90 ± 8.00
	Pedrotti et al,_2020^59^	1	25	3	70.16 ± 4.00
	Kohnen et al,_2019^60^	3	26	3	69.00 ± 8.00
	Paik et al,_2020^52^	3	20	3	60.40 ± 10.00
	Nowik et al,_2022^61^	1	29	6	68.30 ± 10.00
	Palomino-Bautista et al,_2021^48^	2	25	3	73.88 ± 4.00
	Farvardin et al,_2021^45^	1	20	11	63.20 ± 6.00
	Gil et al,_2020^19^	2	20	6	68.20 ± 6.00
Vivity	Fernández-Vega-Cueto et al,_2022^62^	7	30	6	76.70 ± 6.00
	Gundersen and Potvin,_2021^63^	1	40	3–12	59.00 ± 8.00
	Rementería-Capelo et al,_2022^64^	1	25	3	76.70 ± 5.00
	Schallhorn,_2021^65^	2	107	6	68.60 ± 7.00
ATLara	Ganesh et al,_2021^66^	8	30	12	65.40 ± 7.00
	Reinhard et al,_2021^67^	1b	78	1	65.00 ± 9.00

DFVA = defocus visual acuity; IOL = intraocular lens.

### Data Analysis

Defocus VA curves with simulated IOLs were compared across lenses in the same subject and on average across subjects. The average DFVA curves measured with each simulated IOL in the current study were compared with the average DFVA curves of pseudophakic patients implanted with the corresponding IOLs compiled from the literature.

Comparisons were made in terms of cross-correlation and RMSE between the average SimVis and the average literature curves. Because of systematic differences in VA across samples (likely due to differences in age range, pupil size, or testing conditions), an offset was estimated by minimizing the RMSE between curves and was applied to the SimVis curves. This process ensured that comparisons were made in terms of curve shape rather than absolute baseline level. Table 6 shows the vertical offset (in logMAR units) applied to the dataset following the RMSE minimization procedure.

**Table 6:** Vertical Offset (in logMAR Units) Applied to the Dataset Following the RMSE Minimization Procedure

Model	Vertical Offset (logMAR)
PanOptix	0.019
FineVision	0.052
ATLisa	0.131
Symfony	0.047
Vivity	0.039
ATLara	0.152

logMAR = logarithm of the minimum angle of resolution; RMSE = root mean square error.

The following parameters were also computed from the DFVA curves (from SimVis simulations and the reference curves from the literature for parameter 1, and for the SimVis simulations only for parameters 2-4): (1) DOF, calculated as the dioptric range for which the logMAR VA was better than 0.20^68^ (for a range of 0 D to –3.00 D for EDOFs, and 0 D to –4.00 D for trifocals); (2) visual degradation at far; (3) visual benefits at intermediate; and (4) visual benefit at near. Parameters 2-4 and intermediate and visual degradation at far were calculated for each subject by taking the DFVA curve of the generic monofocal IOL as a reference (the monofocal reference was programmed in SimVis using a single temporal coefficient [focal state] corresponding to best distance correction) and subtracting the multifocal VA values for the aforementioned distances.^69^ For session I and session II separately, repeated measures analysis of variance tests and post hoc analysis with Bonferroni correction (normally distributed data) or Friedman tests and post hoc analysis with Tukey–Kramer correction (not normally distributed data) were performed to investigate if the mean VA differences are the same across all 3 IOLs at far, intermediate, and near distances.

## Results

### Experimental IOL Characterization: Real and Simulated IOLs

**Figure 2:**
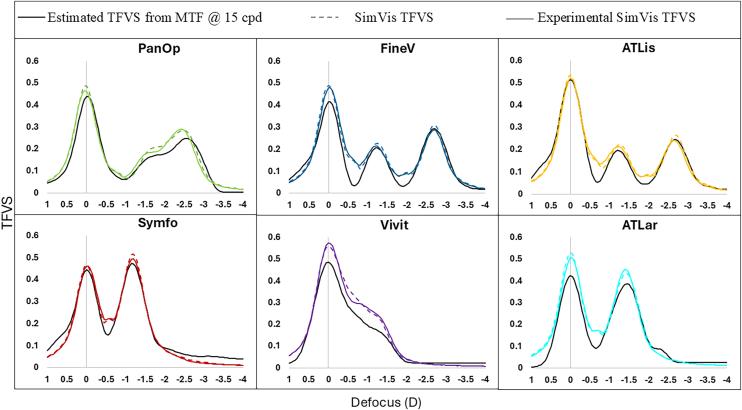
Comparison of the estimated TFVS calculated from the MTF@15cpd (black), the SimVis TFVS (solid-colored line), and the experimental SimVis TFVS (dashed colored line) for the 6 different IOLs. Each color represents one lens: PanOp (green), FineV (blue), ATLis (yellow), Symfo (red), Vivit (purple), and ATLar (turquoise). IOL = intraocular lens; MTF = modulation transfer function; TFVS = through-focus visual Strehl ratio.

Figure 2 presents the on-bench characterization of the real and the simulated IOLs. The estimated TFVS (calculated from reports of the MTF@15cpd measured on real lenses on bench) is presented in black; the estimated SimVis TFVS is presented in colored solid lines; and the Experimental SimVis TFVS (computed from the data obtained with the high-speed focimeter) is presented in dashed colored lines. The simulated IOLs capture, with high degree of accuracy, the TF performance of the real IOLs. For each IOL, the cross-correlation between the curves was >0.982, while the RMSE was <0.040 visual Strehl ratio units.

### Comparison of DFVA Curves with Simulated MIOLs and Literature Reports on Implanted Patients

**Figure 3:**
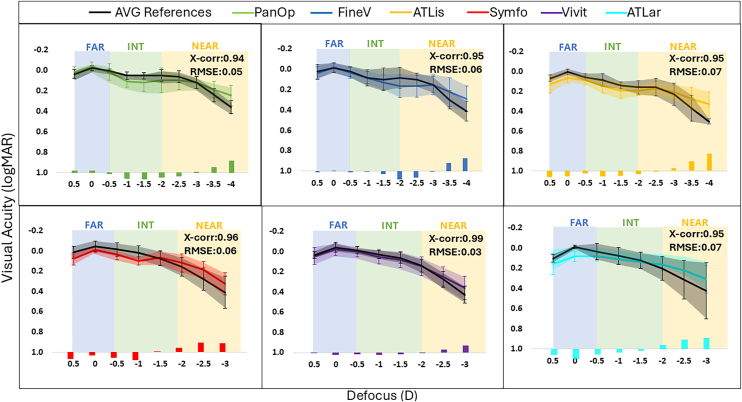
Average DFVA curves with simulated IOL (colored lines) versus average DFVA curves in patients implanted with the corresponding IOL obtained from the literature (black lines), for all tested IOLs. Error bars and shaded areas represent the ±1 standard deviation, and each color represents 1 IOL: PanOp (in green), FineV (in blue), ATLis (in yellow), Symfo (in red), Vivit (in purple), and ATLar (in turquoise). The bars represent the logMAR VA differences between the literature data from implanted patients and the SimVis simulation data for each defocus step (literature data minus SimVis simulation data). DFVA = defocus visual acuity; IOL = intraocular lens; logMAR = logarithm of the minimum angle of resolution; RMSE = root mean square error; VA = visual acuity.

Figure 3 shows the mean DFVA curve in presbyopic subjects with simulated IOLs with SimVis (solid color lines) versus the average DFVA curves in patients implanted with the corresponding IOL obtained from the literature (black lines) for all different IOLs. For clarity of comparison, curves are shown after applying the offset estimated by RMSE minimization and provided in Table 6. This adjustment does not alter the shape of the functions but ensures that the comparisons are based on relative profiles rather than baseline differences. Additionally, the logMAR VA differences between the literature data from implanted patients and the SimVis simulation data for each defocus step (literature data minus SimVis simulation data) are presented as bars in each of the graphs. Error bars represent ±1 standard deviation.

Both the DFVA measured in phakic presbyopic subjects through the simulated lenses and postoperatively in patients with the implanted IOL capture a similar through-focus performance. Differences in the binocular logMAR VA at far are within 0.09 logMAR. The largest discrepancies occur systematically for the largest vergences at near (–2.50 D and beyond), up to 0.17 logMAR for the ATLis at 4 D. The cross-correlation coefficients between the average DFVA with simulated IOL and the average DFVA curve in patients implanted with the IOL were ≥0.94 and the RMSE was ≤0.07 for all IOLs under study.

### DOF, Visual Benefit, and Visual Degradation from DFVA Curves

**Figure 4:**
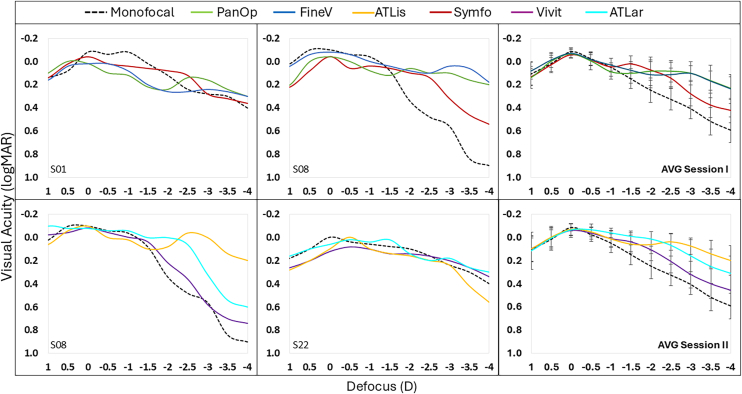
Variability across selected individual subjects for each lens design measured through SimVis Gekko: monofocal (in black), PanOp (in green), FineV (in blue), ATLis (in yellow), Symfo (in red), Vivit (in purple), and ATLar (in turquoise). Top left and top middle graphs show the DFVA curves of Symfo, PanOp, and FineV simulations for S01 and S08, respectively. Bottom left and bottom middle graphs show the DFVA curves of Vivit, ATLis, and ATLar simulations for S08 and S22, respectively. The right panel shows the average DFVA curves for session I (top right) and session II (bottom right). DFVA = defocus visual acuity; logMAR = logarithm of the minimum angle of resolution.

Figure 4 illustrates how through-focus performance across IOLs compares for a single individual. The left and middle panels show the effect of different simulated IOLs on the through-focus visual performance of 4 representative individual subjects as examples. The right panels show averages across the simulated IOLs assessed in subjects in session I (upper graph) or session II (lower graph). There are clear differences in through-focus performance across IOLs. For example, trifocal IOLs (PanOp, FineV, and ATLis) expanded the DOF with respect to the monofocal IOL performance by 1.50 D on average (monofocal DOF 1.50 D; average trifocals DOF 3.00 D). The EDOF IOLs (Symfo, Vivit, and ATLar) expanded the DOF compared with the monofocal IOL by 0.70 D on average (monofocal DOF 1.50D; average EDOFs DOF 2.20 D) and yielded 0.80 D shorter DOF compared with the trifocal designs. However, large intersubject variations across individuals were observed. For instance, subjects with wider monofocal DFVA (such as S01 and S22) showed smaller gains in DOF with EDOF lenses like Symfo and Vivit. A linear mixed-effect regression confirmed that larger baseline monofocal DOF was associated with smaller gains for both trifocal and EDOF designs (trifocals: β = –0.96, *P* < 0.001; EDOFs: β = –0.57, *P* = 0.009). Additionally, the strength of the negative baseline–gain relationship did not differ significantly between trifocal and EDOF groups (interaction *P* = 0.16), supporting that both trifocals and EDOFs showed the same patterns on the added benefit. Adding “session” as a fixed effect did not improve model fit, and no significant differences were observed between session I and session II (*P* = 0.25), indicating that the results were consistent across measurement sessions (no systematic bias). Additionally, subjects with better VA at far under monofocal IOL tended to show greater visual benefit at intermediate and near distances but also experienced greater degradation at far with MIOLs.

**Figure 5:**
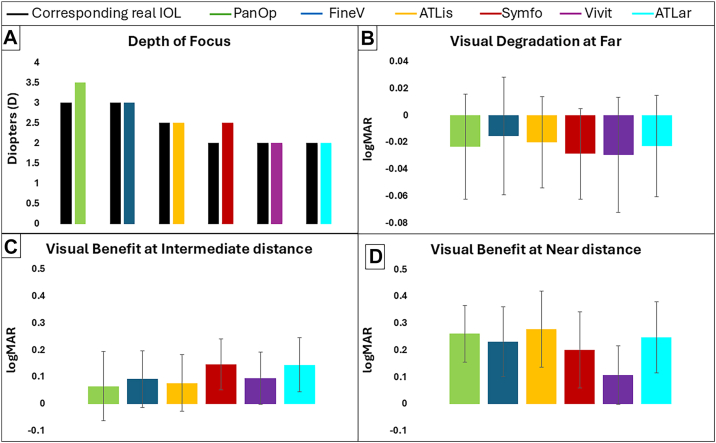
**A,** Depth of focus (DOF) of the SimVis simulation (colored bars) and DOF of the corresponding real IOL (black bars) extracted from the average SimVis and the average literature DFVA curves, respectively. The DOF was calculated as the dioptric range for which the logMAR VA was better than 0.20. **B–D,** The visual degradation at far (0 D) and the visual benefit at intermediate (–1.5 D) and near (–2.5 D) distances, respectively, with each simulated IOL with respect to the monofocal correction (monofocal VA minus MIOL VA); negative difference values indicate better VA for the monofocal IOL, whereas positive difference values indicate better VA for the MIOL. PanOp (in green), FineV (in blue), ATLis (in yellow), Symfo (in red), Vivit (in purple), and ATLar (in turquoise). D = diopter; DFVA = defocus visual acuity; IOL = intraocular lens; logMAR = logarithm of the minimum angle of resolution; MIOL = multifocal intraocular lens; VA = visual acuity.

The DOF with simulated IOLs (color bars) as well as the DOF of the real IOLs (black bars) are presented in Figure 5 (top left graph). In general (in simulations and in real IOLs), trifocals (PanOptix, FineVision, and ATLisa) yielded wider DOF (range 2.50 to 3.50 D) compared to EDOF (Symfony, Vivity, and ATLara) lenses (range 2.00 to 2.50 D). Most of the simulations provided the same DOF as the real IOL; only for Symfony and PanOptix, the simulation provided broader DOF by 0.50 D.

**Figure 6:**
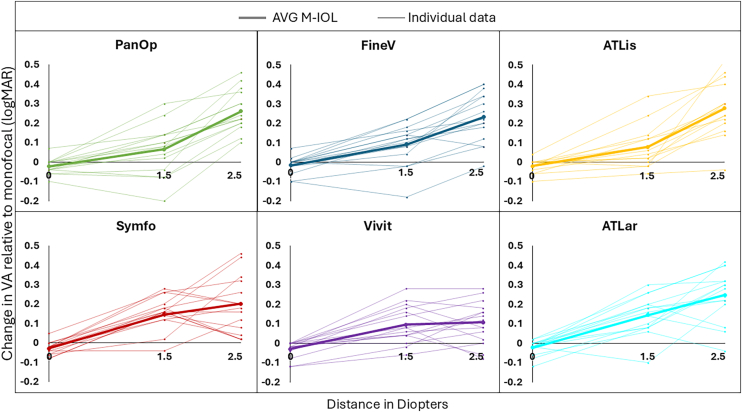
Change in VA relative to monofocal IOL performance (monofocal VA minus MIOL VA) for 3 distances (0 D for far, 1.50 D for intermediate, and 2.50 D for near vision) for each simulated IOL; negative difference values indicate better VA for the monofocal IOL, whereas positive difference values indicate better VA for the MIOL. PanOp (in green), FineV (in blue), ATLis (in yellow), Symfo (in red), Vivit (in purple), and ATLar (in turquoise). Individual data are represented by thin lines and the averages by thick lines. IOL = intraocular lens; logMAR = logarithm of the minimum angle of resolution; MIOL = multifocal intraocular lens; VA = visual acuity.

The visual benefit at intermediate (–1.50 D) and near (–2.50 D) distances, as well as the visual degradation at far (0 D), were calculated for each subject and the average values across subjects, along with intersubject variability (shown as error bars), are presented in Figure 5. Although on average all MIOLs degraded VA at far compared with the monofocal IOL (degradation ranged from –0.029 to –0.015 logMAR), they improved VA at intermediate (improvement ranged from +0.065 to +0.147 logMAR) and near (improvement ranged from +0.108 to +0.277 logMAR) distances. Among trifocal IOLs, ATLis showed a slightly higher improvement at near compared with PanOp and FineV. For EDOF IOLs, Symfo showed the largest VA gain for intermediate distance and ATLar for near distance. Figure 6 presents the individual data (thin lines) of visual benefit and degradation, as well as the average (thick lines) plotted across the 3 distances. Trifocal IOLs exhibited steeper improvements in VA at near distance compared to EDOFs, consistent with their optical design intent. Vivit exhibited the flattest slope, indicating more limited benefit beyond intermediate distance. Individual variability was present across all IOLs, with a general increase in dispersion at 2.50 D, particularly for Symfo and ATLis, whereas FineV exhibited less variability in VA at near. A large variance can be observed in the data. In terms of IOL categories, EDOF and trifocal IOLs showed similar average variance in VA at far (0.015 logMAR) and near (0.016 logMAR) distances, whereas for intermediate distance, EDOFs showed a smaller variance compared to trifocal IOLs (0.009 and 0.013 logMAR, respectively). The outcomes of the post hoc comparisons for sessions I and II at far, intermediate, and near distances are summarized in Table 7. In session I, no significant differences were found at far or near distances, whereas Symfony provided significantly greater visual benefit than FineVision at intermediate distance. In session II, no differences were found at far distance; ATLara provided greater benefit than ATLisa at intermediate distance, and both ATLisa and ATLara provided greater near benefit than Vivity.

**Table 7:** Summary of Post Hoc Comparisons across IOLs

Session	Distance	Significant Comparison	Direction of Effect	*P* Value
I (Symfo, PanOp, FineV)	Far	None	-	0.11
I	Intermediate	Symfo vs. FineV	Symfo better	0.003
I	Near	None	-	0.10
II (Vivit, ATLis, ATLar)	Far	None	-	0.58
II	Intermediate	ATLar vs. ATLis	ATLar better	0.008
II	Near	ATLis vs. Vivit	ATLis better	0.001
II	Near	ATLar vs. Vivit	ATLar better	0.00009

IOL = intraocular lens.

## Discussion

This study demonstrates the feasibility and clinical relevance of using visual simulators (specifically the SimVis Gekko binocular simulator, based on temporal multiplexing technology) to reproduce and compare the through-focus visual performance of 6 commercially available MIOLs in the same presbyopic individuals.

We have implemented a robust, validated method for programming the SimVis Gekko to simulate commercially available MIOL designs using publicly available data. Specifically, temporal coefficients necessary for temporal multiplexing were computed from bench-measured TF-MTF curves. This process allows clinicians and researchers to evaluate actual commercial MIOLs preoperatively. While very robust, this process has some limitations. Simulations were performed using a fixed 3-mm pupil, whereas in clinical practice natural pupil size varies among patients and conditions. Additionally, while the simulator is capable of capturing the lens optical behavior, it cannot replicate surgical factors (e.g., lens decentration, capsular contraction, or incision effects) or design features outside the optical zone (e.g., haptics and edge design), all of which may influence postoperative outcomes.^70^^,^^71^ The interaction between the subject's native aberrations and the simulated lens design also presents a source of variability that merits further exploration.

An important factor to consider when interpreting the simulated DFVA curves is the potential contribution of residual accommodation in phakic presbyopic subjects. Although accommodation is substantially reduced in presbyopia, several studies have reported residual accommodative amplitudes in the order of 0.5-1.0 D,^72^ which may contribute to improved visual quality at near distances during simulation-based measurements. This effect is particularly relevant at higher near vergences, where the simulated DFVA curves consistently show slightly better performance than the postoperative DFVA curves extracted from the literature. In this context, residual accommodation, together with neural DOF and adaptation to defocus, may partially explain the enhanced near VA observed with SimVis simulations, especially beyond the nominal add range of the IOL designs. This observation suggests practical implications, especially regarding whether accommodation should be paralyzed during simulations, at least in early presbyopes, or whether crystalline lens contributions should be controlled through other means.

The presence of residual accommodation may also explain the bimodal shape observed in some of the monofocal DFVA curves obtained in this study. A secondary improvement in VA at intermediate vergences may arise from accommodative responses or from the combined effects of residual accommodation and neural tolerance to defocus. Importantly, these physiological factors are expected to affect all simulated lens conditions similarly within a given subject and therefore do not compromise the comparative validity of the simulations across different IOL designs.

The simulated DFVA curves obtained in 23 presbyopic subjects strongly matched published postoperative DFVA data for each of the 6 MIOLs. We found RMSE of 0.06 and a cross-correlation coefficient of 0.95 when comparing simulated DFVA curves to those reported in the literature for the FineVision IOL, closely aligning with the values from previous studies (Vinas et al reported an RMSE of 0.05 and *r* = 0.955 for simulations of a trifocal IOL).^29^ This agreement across independent studies supports the reliability and validity of the simulation process. Moreover, we benchmarked against a broad pool of literature data, providing a more representative comparison. Still, differences in methodology remain. For example, the participating subjects used fixed pupils, while postoperative data presents results with natural pupils. Additionally, our subjects retained crystalline lenses, unlike pseudophakic patients in literature. These differences may account for small discrepancies and should be considered when interpreting results.

The comparative analysis of DFVA curves across lenses yielded new insights into how different IOL designs impact the DOF. The Symfony lens, for example, produced a flatter DFVA curve and shorter DOF, in line with its classification as an EDOF lens. In contrast, lenses like FineVision and PanOptix showed greater VA at near and intermediate distances but more degradation at far, reflecting their multifocal design characteristics. These findings confirm that the SimVis technology captures clinically meaningful differences in visual performance among IOLs. By analyzing the simulated DFVA curves with our new metrics, we have enhanced the understanding of lens behavior across different visual distances, facilitating informed choices tailored to a patient's needs and lifestyle.

The present validation relies on bench-derived TF-MTF data obtained under controlled optical conditions. Differences in measurement parameters across sources including monochromatic versus polychromatic illumination, pupil diameter, and the handling of spherical aberration, may influence the shape of the reported optical profiles and consequently the simulated DFVA curves.^25^^,^^42^ In this study, polychromatic weighting was applied when wavelength-specific data were available; however, residual discrepancies between bench measurements and clinical VA are expected, given that VA integrates information across spatial frequencies and is influenced by neural factors.^73^ While MTF at a single spatial frequency (15cpd) has been shown to correlate well with VA,^74^ alternative metrics incorporating multiple spatial frequencies or visual Strehl-based approaches may further improve prediction accuracy or reduce interstudy variability and warrant future investigation.

Another limitation of the present work is that the validation was performed in subjects with clear crystalline lenses. Previous studies have shown that preoperative temporal multiplexing simulations correspond well with postoperative visual outcomes in patients undergoing cataract surgery with presbyopia-correcting IOLs.^30^^,^^75^ Such clinical studies indicate that preoperative simulations may be useful in predicting individual postoperative performance and supporting IOL selection, but the current study was not designed to replicate within-subject preoperative versus postoperative comparisons in cataract population. Therefore, while these findings are promising for clinical translation, caution is needed when applying the present results to cataract patients, especially those with advanced lens opacification. Ongoing work will further investigate preoperative simulation and postoperative outcomes within the same eyes across a range of IOL designs and clinical profiles with the aim of providing stronger evidence of predictive performance in surgical practice.

A key strength of our study is the ability to assess variability in visual performance with the same lens design across multiple individuals. Interestingly, some individual cases reached performance levels exceeding the monofocal IOL at all vergences, while others performed worse, highlighting the importance of patient-specific outcomes. Average performance obtained from literature data appears to mask some of this individual variability, especially in lenses like PanOp and ATLis, where the group mean suggests steady gains, but individual curves reveal that some subjects had negligible or even negative benefits at intermediate. The standard deviations in the VA measured with simulated lenses (0.11 averaged across distances, subjects, and lenses) are comparable to the reported standard deviations postoperatively in patients implanted with these IOLs. This suggests that SimVis Gekko captures both the average and the individual visual performance with good accuracy. Intersubject differences likely arise from individual optical aberrations, pupil size, and neural factors and cannot be fully predicted from preoperative metrics alone. This demonstrates the value of visual simulators in facilitating IOL selection based on each patient's unique visual system.

One of the most clinically relevant advances of this study is the ability to simulate and compare multiple MIOLs in the same subject, something that is not possible postoperatively. In this cohort, differences in DFVA curves were observed not only between lens categories, but between EDOF IOLs and between trifocal IOLs, in the same individual. The experimental simulations also reveal differences in perceived visual quality that may not be evident from optical bench tests or averaged clinical data. Although our results do not yet establish predictive criteria for IOL selection based on preoperative vision alone, they clearly highlight the limitations of conventional assessment.

## Conclusion

This study demonstrates that multiple commercial MIOLs can be accurately simulated in a preimplantation context using SimVis Gekko. The strong alignment of simulated and postoperative DFVA curves validates the ability of SimVis technology to simulate visual performance under natural viewing conditions. Our findings show that SimVis Gekko captures differences across lens types and across individuals, supporting its use as a preoperative decision-making tool.
